# Accuracy of phylogeny reconstruction methods combining overlapping gene data sets

**DOI:** 10.1186/1748-7188-5-37

**Published:** 2010-12-06

**Authors:** Anne Kupczok, Heiko A Schmidt, Arndt von Haeseler

**Affiliations:** 1 Center for Integrative Bioinformatics Vienna, Max F. Perutz Laboratories, University of Vienna, Medical University of Vienna, University of Veterinary Medicine Vienna, Dr. Bohr-Gasse 9, A-1030 Vienna, Austria; 2 Current Address: IST Austria, Am Campus 1, A-3400 Klosterneuburg, Austria

## Abstract

**Background:**

The availability of many gene alignments with overlapping taxon sets raises the question of which strategy is the best to infer species phylogenies from multiple gene information. Methods and programs abound that use the gene alignment in different ways to reconstruct the species tree. In particular, different methods combine the original data at different points along the way from the underlying sequences to the final tree. Accordingly, they are classified into superalignment, supertree and medium-level approaches. Here, we present a simulation study to compare different methods from each of these three approaches.

**Results:**

We observe that superalignment methods usually outperform the other approaches over a wide range of parameters including sparse data and gene-specific evolutionary parameters. In the presence of high incongruency among gene trees, however, other combination methods show better performance than the superalignment approach. Surprisingly, some supertree and medium-level methods exhibit, on average, worse results than a single gene phylogeny with complete taxon information.

**Conclusions:**

For some methods, using the reconstructed gene tree as an estimation of the species tree is superior to the combination of incomplete information. Superalignment usually performs best since it is less susceptible to stochastic error. Supertree methods can outperform superalignment in the presence of gene-tree conflict.

## Background

The phylogenetic information inherent in sequence data from different genes can be combined to yield a species phylogeny rather than gene trees. The gene data for these phylogenies are mainly collected following two strategies: (a) using only genes that provide full information, i.e., cover all taxa of interest (e.g. [1]) or (b) using all available genes that are present in some taxa and fulfill special overlap conditions (e.g. [2-4]). The latter approach is able to use many more genes and taxa, since it allows for missing data. It can also be applied for phylogeny reconstruction from expressed sequence tags (ESTs, e.g. [5]). Before the gene alignments are obtained, two important steps can influence the phylogeny result: First, orthologs must be assigned correctly (see e.g. [6,7] for method comparisons). Second, these orthologs need to be aligned with sufficient accuracy (see e.g. [8] for a review and [9] for an example of the impact of alignment accuracy on phylogeny reconstruction).

After reliable alignments are obtained, different methods are available to combine the original data at different points along the way from the underlying sequences to the final tree [4,10]: First, superalignment methods combine the data at an early level by directly concatenating the gene alignments without any intermediate computations (early-level combination; also called "supermatrix", "concatenation" or "total evidence" [11,12]). Superalignment methods have been used to infer phylogenies for eukaryotes [13], Metazoa and green plants [2], legumes [3] or species from all three domains of life [1].

Second, medium level combination methods first compute intermediate results from the gene alignments, e.g. pairwise distances [14,15] or quartets [4], and subsequently reconstruct a phylogeny by combining this information.

Third, supertree methods combine the data at the late level of gene trees (late-level combination; e.g. [16]). Gordon [17] first suggested supertree methods to combine overlapping trees. The so-called source trees are first computed for each gene, or are obtained from the literature, and are subsequently combined into a supertree. The prevalent method for reconstructing supertrees is matrix representation with parsimony (MRP) [18,19], especially when only published trees but not the original data are available or when data of different kind are combined. MRP has been applied to many different kinds of species data, for instance to Mammalia [20] or Bacteria [21].

Each of these approaches has general advantages and disadvantages. The superalignment method uses all character information but assumes the same underlying topology and often the same parameters for all genes. Supertree approaches account for differing topologies and parameters between genes. On the other hand, they are more susceptible to stochastic errors since estimating substitution parameters and a topology for each gene independently may lead to overfitting. Furthermore, they try to minimize the amount of missing data when constructing the gene trees. Medium-level approaches also allow for gene-specific parameters, but they use quartet likelihoods or distances, not gene trees, when building the final tree. In the consensus setting, i.e., where all data sets contain the same taxa, the differences between concatenated alignments and tree combination have been extensively discussed (e.g. [22-27]).

Practical investigations using real data sets or simulated data are of interest to compare different methods. Various authors used real data sets to compare superalignment and supertree approaches [7,28-31]. Those real data sets have the advantage of a realistic setting, however, the true tree is usually unknown. Then it is only possible to use well-established clades for assessing the performance (e.g. [7]) or to compare methods to one another (e.g. [31]). In simulations, on the other hand, the results can be compared to a model tree. Then the performance of the methods can be measured at an absolute scale. Several studies investigating supertree methods using simulations were carried out [32-35]. They employed the following general scheme: (1) Generation of a model tree assuming a Yule process, (2) generation of alignments along that tree, (3) random deletion of a proportion of taxa, (4) reconstruction of gene trees by maximum parsimony, (5) construction of the supertree from the inferred gene trees, and (6) comparison of the supertree to the model tree. Bininda-Emonds and Sanderson [32] compared superalignment and MRP for different degrees of divergence and observed that, with increasing divergence, the distance of the MRP trees to the superalignment tree increased. Levasseur and Lapointe [35] compared average consensus, superalignment with distances and MRP for gene trees with complete taxon sets. They found average consensus to perform nearly as well as superalignment, whereas MRP was substantially worse since it ignores gene tree branch lengths.

Simulations can also be used to evaluate the impact of undesired properties for a particular supertree method. For instance, one of these properties is the emergence of "novel clades", i.e., clades contradicted by all gene trees. Bininda-Emonds [33] found such clades to be very rare. However, note that due to missing taxa and multifurcating trees, it is not straightforward to measure supporting and conflicting relationships between a supertree and the gene trees (an alternative definition is presented in [36]).

Each of the above simulation studies focused on a special subset of methods for supertree construction. A general performance assessment, however, has not yet been carried out, and the strengths and weaknesses of the different methods are unknown. Here, we present an extensive simulation study about combining gene alignments. Thus, we take the orthology relationships and the alignment as correctly given. We compare different data combination methods, including supertree, superalignment and medium-level methods, to assess their accuracy in biologically reasonable situations. This leads to suggestions of applicable methods in the case of overlapping data sets. Moreover, we discuss the issue of complete versus incomplete data.

## Methods

### Phylogenetic Reconstruction from Multiple Data Sets

We evaluate a list of methods spanning the range from early- to latel-level combination. All methods investigated, together with the abbreviations used, are listed in Table 1.

**Table 1:** Overview of reconstruction methods and corresponding abbreviations

Abbreviation	Description	Reference	
**Late-level combination:**			

Consensus	Majority-rule consensus	[83]	
MRP_BR	Matrix representation with parsimony and Baum/Ragan coding	[18,19]	
MRP_PU	Matrix representation with parsimony and Purvis coding	[43]	
MRP_I	Matrix representation with irreversible parsimony and Baum/Ragan coding	[46]	
MRF_BR	Matrix representation with flipping and Baum/Ragan coding	[47,48]	
MRF_PU	Matrix representation with flipping and Purvis coding	-	
MRC	Matrix representation with compatibility and Baum/Ragan coding	[50,51]	
MinCut	Minimal cut	[54]	
ModMinCut	Modified minimal cut	[55]	
MaxCut	Maximal cut	[57]	
QILI	Quartet inference and local inconsistency	[58]	

**Medium-level combination:**			

SuperQP	Super quartet puzzling	[4]	
AvCon	Average consensus	[14,63]	
SDM	Super distance matrix	[15]	

**Early-level combination:**			

SA	Superalignment	e.g. [11]	

#### Early-level combination

A superalignment is generated from single gene alignments by concatenating the different alignments and adding gaps where no sequence information is present for a specific taxon. The superalignment method (**SA**) refers to reconstructing the superalignment tree. Here, we use maximum likelihood (ML) or maximum parsimony (MP), depending on the size of the data set. ML phylogenies are computed with IQPNNI version 3.1 [37], assuming the substitution model HKY for DNA sequences [38] and JTT for protein sequences [39]. In both cases, site heterogeneity is modeled with four -distributed rate categories. MP phylogenies are computed with PAUP* 4.0b10 [40] and the following parameters: heuristic search with TBR branch swapping, random addition of sequences, and a maximum of 10,000 trees in memory.

#### Late-level combination

##### Phylogenetic reconstruction of gene trees

**Figure 1:**
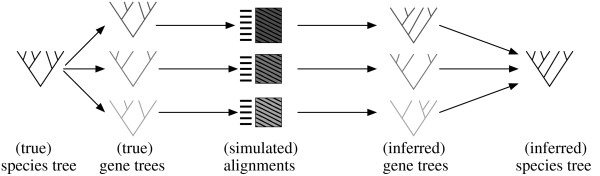
**Diagram of the simulation setting with supertree reconstruction**. The simulation proceeds in several steps: First, gene trees are generated from the given species tree. Alignments are simulated along these gene trees. From these alignments gene trees are inferred. The inferred gene trees are the source trees for supertree reconstruction. With the supertree methods species trees are inferred.

The first step of any late-level combination method is the reconstruction of the gene phylogenies (Figure 1), which serve as source trees for the supertree reconstruction. We compute ML gene trees with IQPNNI using the same reconstruction parameters as for the early-level combination. In some simulations, the gene trees are obtained via bootstrapping. In this case, we generate 100 bootstrap replicates of each gene alignment with seqboot, compute phylogenies with IQPNNI and subsequently build a majority-rule consensus tree of the bootstrapped trees for each gene with consense. Both seqboot and consense are part of PHYLIP version 3.6 [41].

##### Consensus

For complete data, where each gene is present in each taxon, we also apply the majority-rule consensus as implementated in consense.

##### Methods Using Matrix Representation

Three methods based on matrix representation (MR) coding schemes are available: MR with parsimony (MRP), MR with flipping (MRF), and MR with compatibility (MRC). All three aim to optimize an objective function. If more than one optimal tree is found, we take the strict consensus tree as the reconstructed tree.

Different coding schemes have been suggested to decompose the gene trees into an MR: In the Baum-Ragan (**BR**) coding scheme, every gene tree topology is coded as follows [18,19,42]: An interior edge in a tree divides the taxa into two disjoint sets. For each interior edge, a column is added to the MR, where '0' and '1' indicate the taxa on either side of the edge and missing taxa are coded as '?'. For rooted trees, the root-side is always coded as '0'. The Purvis (**PU**) coding scheme can only be applied to rooted trees. Then, sister groups are coded binarily, and the remaining taxa are coded as '?' (see Table 2 for an example). This aims at removing some redundant information [43]. We generate both matrix representations from the list of gene trees using r8s version 1.71 [44].

**Table 2:** Example of coding a gene tree as a matrix representation

Tree	Baum/Ragan coding	Purvis coding
	A 11 B 00 C 10 D 11	R 00 A 11 B 0? C 10 D 11

The Baum/Ragan coding codes every internal split independently. We use the unrooted version of the BR coding, i.e., without coding the root explicitly. The Purvis coding codes only sister groups of rooted trees.

**MRP **trees are reconstructed by searching the most parsimonious tree for the matrix representation [18,19,42]. We apply two kinds of parsimony: (1) reversible Fitch parsimony [45], which assumes the character changes to be undirected, and (2) irreversible Camin-Sokal parsimony, which only allows changes from 0 to 1 and thus uses the root information in the trees [46]. The most parsimonious tree with the respective criterion is determined by PAUP* 4.0b10 (heuristic search with TBR branch swapping and random addition of sequences, and a maximum of 10,000 trees in memory). Overall, we consider three MRP variants: MRP_BR (reversible parsimony and BR coding), MRP_I (irreversible parsimony and BR coding) and MRP_PU (reversible parsimony and PU coding).

The objective function of **MRF **is to minimize the number of binary flips (changes from '0' to '1' and vice versa) necessary to convert the original MR into an MR compatible with a tree [47,48]. Here, we apply MRF to both coding schemes, BR and PU. So far, MRF has only been applied to matrices with Baum/Ragan-coding. Since MRF, like MRP, is an NP-complete problem, we use the heuristic implemented in HeuristicMRF2 (http://genome.cs.iastate.edu/CBL/[49]).

The objective of **MRC **is to maximize the number of columns in the MR congruent with a tree [50,51]. We use Clann version 3.0.2 as a heuristic to find the MRC tree for a BR coded matrix representation (the sfit criterion with default parameters [52]).

##### Variants of the "Build" algorithm

The "Build" algorithm [53] is only able to construct a supertree for a set of compatible and rooted gene trees. In case of compatible gene trees, each gene tree is a subtree of the supertree. "Build" and its variants are graph-based rooted triplet methods, thus, rooted trees are required. To combine incompatible gene trees, different **cut methods **have been developed.

**MinCut **(minimal cut) is an extension of the "Build" algorithm [54]. In case of a conflict, MinCut introduces an edge in the supertree that conflicts with the fewest possible number of triplets.

**ModMinCut **(modified MinCut) improves MinCut by not only considering the contradicting triplets for an edge but, additionally, by trying to keep subtrees that are uncontradicted by the gene trees [55]. Both MinCut and ModMinCut are polynomial-time algorithms implemented in supertree by Rod Page. We use a precompiled version of this program taken from Rainbow 1.2 beta [56].

**MaxCut **[57] considers two types of triplet topologies: bad ones which occur in a gene tree, and good ones for which another possible topology occurs in a gene tree. In case of a conflict, the ratio of these counts is maximized, which is an NP-hard problem. Snir and Rao [57] suggested a heuristic based on semidefinite programming. We compute the MaxCut tree from a set of triplets with a program provided by Sagi Snir. To apply it, we first extract triples from the gene trees using a program provided by Gregory Ewing.

**Quartet-based methods QILI **(Quartet Inference and Local Inconsistency) [58] is based on quartet topologies extracted from unrooted gene trees. First, a set of weighted quartets is computed, where the weights for each quartet are smaller if they occur in more trees. Missing quartets are inferred by a rectifying process using quintet information. From this collection of quartets, a tree is estimated by minimizing the weighted sum of the quartets represented in a tree using Willson's local inconsistency method [59]. QILI is available in the QuartetSuite 1.0 package.

#### Medium-level combination methods

##### Quartet-based methods SuperQP

combines the sequence data based on the quartet likelihoods [4]. For each gene, TREE-PUZZLE [60] computes all quartet tree likelihoods. These likelihoods are combined for every possible quartet topology across all genes containing the respective quartet. The likelihoods are used to combine the data into so-called superquartets, the building blocks for SuperQuartetPuzzling (SuperQP). SuperQP is related to the QP algorithm [61], but it takes also missing data into account, using an overlap-graph guided insertion scheme and a voting procedure that is aware of missing quartets. We compute the SuperQP tree with an upcoming version of the TREE-PUZZLE package.

##### Distance-based methods

The medium-level information for distance-based methods are pairwise distance matrices computed separately for each gene. Here, we estimate pairwise ML distances with IQPNNI. The distances are combined into one distance matrix for all taxa, which is subsequently fitted to a tree with the least-squares method of Fitch-Margoliash [62]. We use the fitch implementation in the PHYLIP package with the Subreplicates option, thus allowing for missing data by considering only available entries. Two distance-based medium-level methods, differing only in the combination of the matrices, have been devised so far:

With average consensus (**AvCon**) each entry of the combined distance matrix is computed by averaging over all distances available for the corresponding pair of taxa [14,63].

Super Distance Matrix (**SDM**) [15] inserts two types of parameters: (1) weighting factors for each distance matrix, which correspond to a branch lengths scaling for each gene tree, and (2) additive constants for each taxon in each matrix, which correspond to an elongation of terminal branches. Utilizing several contraints, the variance of the scaled and shifted gene distance matrices to the combined distance matrix is minimized. Both methods are impemented in the SDM program [15].

### Simulation Setting

#### Parameters

Figure 1 gives an overview of the simulation setting and notations. We study different parameters involving the underlying data set, the coverage of the sequence data, the topology and parameters of the true gene trees and the sequence lengths (Table 3). The last three parameters will be described in detail along with the results.

**Table 3:** Parameters varied in the simulations

Parameter	Options	
Data set	**S**:	small
	**L**:	large

Taxa coverage	**c**:	complete
	**m**:	missing

True gene trees	**E**:	subtrees of species tree
	**R***_α_*:	rate of evolution assigned randomly from a Γ-distribution with parameter *α *(i.e., mean 1 and variance 1/*α *)
	**P**:	substitution parameters and branch lengths gene-specific
	**G**:	trees gene-specific
	**T***_θ_*:	trees random by coalescent process with parameter *θ*

Reconstructed	**e**:	equal to true gene trees
gene trees	**n**:	normal sequence length and ML estimation

The setting in each simulation is abbreviated by one of the bold letters given in each of the four categories.

Note that not all combinations were tested.

**Figure 2:**
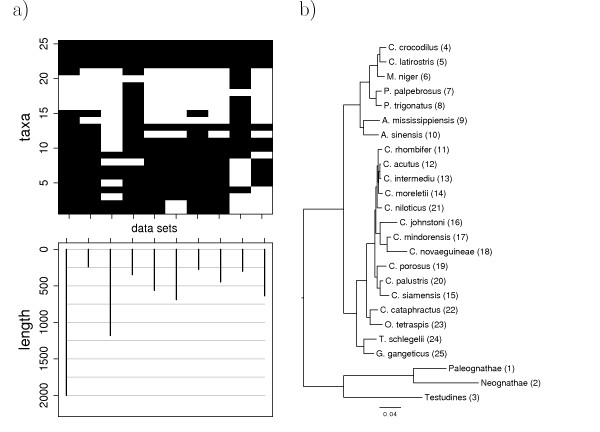
**Small data set (crocodile data)**. a) Distribution of taxa and gene length in the 10 data sets. On average, 65.2% of the genes are present in a taxon. b) Superalignment ML tree. The numbers in brackets refer to taxa numbers in the axis of a).

Like Salamin et al. [64] and Gadagkar et al. [27], we simulate according to biologically reasonable assumptions by taking simulation parameters from real data. We use two data sets:

**The small data set **is given by the parameters of the crocodile data of Gatesy et al. [29]. This data consists of 10 DNA alignments, morphological traits, two RFLP matrices, two allozyme data sets, chromosomal morphology and nest type information for a total of 86 recent and extinct crocodile taxa. Here, we only use the DNA data, which reduces the taxon set to 25 recent taxa and a superalignment of 6,681 sites. Our reconstruction of two superalignment ML trees, one with HKY + Γ and one with GTR + Γ, results in the same tree topology but different branch lengths (HKY tree in Figure 2b). This topology is more resolved than the one by Gatesy et al. [29], and in addition, there is one resolution conflicting with the superalignment tree computed by Gatesy et al. [29]: in our analysis, *C. palustris and C. siamensis *form a clade instead of *C. porosus *and *C. palustris*. We use the HKY tree (Figure 2b) as the species tree for subsequent simulations. For methods requiring rooted gene trees, we root each tree artificially with a taxon in which all genes are present (*O. tetraspis*, taxon 23). Such a procedure was suggested by Baum [18]. Thus the small data set contains of 25 taxa and 10 genes having different sequence lengths and taxa occurences (Figure 2a). Furthermore, the species tree shows a highly non-uniform branch length distribution (Figure 2b). These features are typical for real data sets.

**Figure 3:**
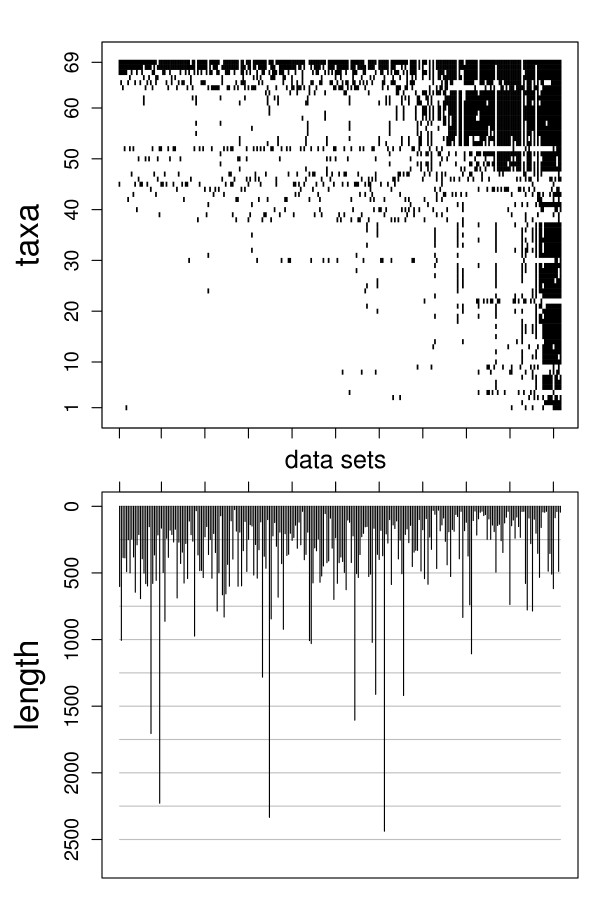
**Large data set (green plant data)**. Distribution of taxa and gene length in the 254 data sets. On average, 15.8% of the genes are present in a taxon.

**The large data set **is composed of 254 proteins from 69 green plants with an overall length of 96,698 amino acids [2]. Driskell et al. [2] describe this data set as problematic, since their reconstructed tree shows relations not supported by any gene tree and the numbers of supporting genes seem to be barely correlated with the bootstrap support for clades. The data contain a higher fraction of missing data compared to the small data set (Figure 3). As species tree we use the superalignment ML tree of the original data, reconstructed with the JTT substitution matrix. Since the data contain no taxon for which all genes are available, every reconstructed gene tree is rooted at the edge that best matched the true rooting. Thereby the model tree is rooted with the taxon suggested in [2].

#### Sequence simulation

For most simulations, the superalignment ML tree for the real data is taken to be the true species tree. Estimated nucleotide and amino acid frequencies as well as the parameter of the -distribution are used as parameters for Monte-Carlo simulations with seq-gen[65]. Unless stated otherwise, protein data are generated with JTT and nucleotide data with an HKY model with the transition/transversion ratio taken from the original ML estimation. Sequences are simulated with the same lengths distribution as in the original data. If simulations were performed taking missing taxa into account, those taxa were deleted from the genes which were also absent in the original data.

There is also the possibility to use the gene trees from the original data as the true gene trees (true gene trees gene-specific, G in Table 3). In this case there is no true species tree known.

For each simulated data set, at most fifteen different methods are applied to reconstruct a tree (Table 1). Note that not all methods are applicable for all settings. Consensus is only applicable for complete data and the medium- and low-level methods are only applicable if sequence information is present.

#### Tree Distance Computation

If applicable, we measured the accuracy of the methods by the normalized Robinson-Foulds distance (RF) of the inferred species tree to the true species tree. The Robinson-Foulds distance [66] is the number of splits that are present in one tree but not in the other one, and vice versa. Since unrooted *n*-taxa trees have a maximum of *n *- 3 inner branches, the maximal Robinson-Foulds distance is 2(*n - *3). In the following, *RF *denotes the *normalized *Robinson-Foulds distance, where the distances are divided by 2(*n *- 3). This yields a value between 0% and 100%, which can be interpreted as the percentage of false or missing splits in the inferred tree compared to the true tree.

## Results and Discussion

Each simulation setting is abbreviated by four letters corresponding to values for each of the four categories of simulation parameters (Table 3).

### Complete data (S, c, E, n)

**Figure 4:**
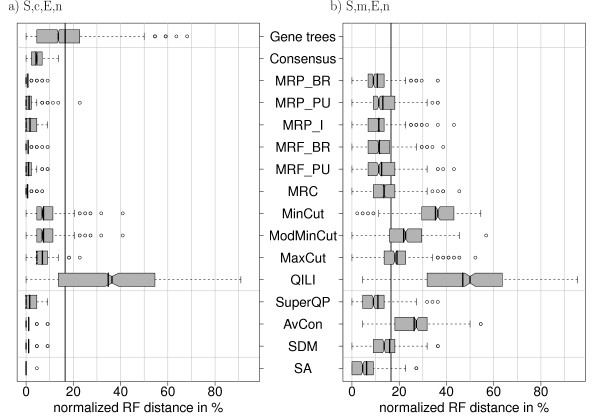
**Distribution of normalized RF distances (500 simulations) for the simulation settings S, c, E, n and S, m, E, n**. The reconstructed trees were compared with the model tree via the RF distance (see methods for details). The distributions resulting from 500 repetitions are shown. The boxes mark the 1/4- and 3/4-quantiles, the vertical line with the notches is the median with the 95% confidence intervall for comparing two medians. The vertical line without the notches is the mean of the data. The vertical black line drawn throughout the diagrams is the mean *RF *distance of all complete gene trees, which serves as the baseline distance.

The first and simplest simulation is that the topology and parameters of the species tree equal those of the true gene trees and the length of each gene alignment is taken from the original data set. In 500 replications, SA nearly always reconstructs the true tree, i.e., *RF *= 0 (Figure 4a). The MR methods and the intermediate methods show mean *RF *distances of less than 2%. In contrast, the mean distance of an inferred single gene tree to the true species tree is 16.5%. This value can be viewed as the mean distance when reconstruction is based on the sequence information of one gene only. Therefore we will call it the *baseline distance*. Surprisingly, QILI shows a mean RF distance of 35%, which is much larger than 16.5%. Thus, accuracy is lost by combining gene trees with this method.

### Missing data (S, m, E, n)

Next, we use the same 500 simulated alignments as before, but delete those sequences from the simulated gene alignments which are not present in the original alignment (cf. Figure 2a). The resulting distributions of the *RF *distances (Figure 4b) show that all methods are strongly affected by missing data. With a mean *RF *distance of about 6.2%, SA is again the most accurate method. Among the remaining methods, MRP_BR (10.8%) and SuperQP (11%) show the smallest mean *RF *distances. The cut methods, QILI, and average consensus show mean RF distances larger than the baseline distance of 16.5%. Thus, these methods perform on average worse on incomplete data sets than the ML reconstruction using only one gene present in all taxa. These methods seem to be unable to efficiently utilize the additional information provided by extra, but incomplete, gene data.

### Large data set (L, m, E, n)

**Figure 5:**
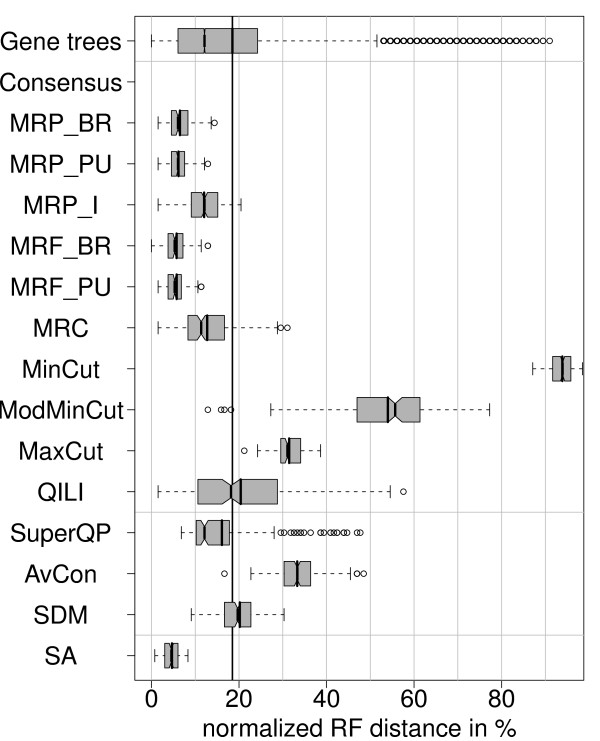
**Distribution of normalized RF distances (200 simulations) for the simulation setting L, m, E, n**. Large data set with missing data according to Figure 3. "Gene Trees" shows the distances of the trees from the complete alignments, not from the pruned alignments, although the latter are used for the data combination methods.

This simulation uses the data set of 254 genes from 69 green plant species (see method section). Compared to the small data set, the alignment of the large data set contains more taxa, more genes, but a smaller fraction of genes present per taxon (Figure 3). Here, we study the simplest simulation setting with missing data. Although SA trees are reconstructed with parsimony to keep computing time reasonable, they still show the highest accuracy with a mean *RF *distance of 4.8% (Figure 5). Among the MR methods, MRP_I (12%) is no longer as accurate as the other MR methods. MRF_BR (5.7%) and MRF_PU (5.8%) are the supertree methods with the highest accuracy. MinCut (93.9%) reconstructs trees that are very distant to the true species tree. A possible reason is the high proportion of missing data. The accuracy of MinCut is improved by ModMinCut (54%) and MaxCut (31.5%), but all cut methods show larger distances than the average complete gene tree (the baseline distance, 18.5%). QILI shows a much better performance compared to the small data set, its mean accuracy (20.4%) is now comparable to SuperQP (16.1%) and SDM (20.2%). These methods show average distance values very close to the baseline distance. But QILI still has a high variance, whereas SuperQP shows good results in most cases and produces unresolved trees in a few cases.

In general, the results of the large data set are similar to those for the small data set: In both settings, the methods that improve the baseline distance are the same, superalignment outperforms the other methods, the MR methods are the best supertree methods, and SuperQP is the best medium-level method. Thus, we expect the results also to be similar when introducing deviating settings. In the following, we only present the results for the small data set.

### Long sequences (S, m, E, l)

We also test whether the methods are able to combine highly informative, but incomplete, data sets. Thus, we minimize the effect of erroneous gene tree reconstruction by generating gene sequences ten times longer than the original gene sequences while taxa occurrences are the same as in Figure 2a. The accuracy of inferred species trees and gene trees is substantially improved for all methods (data not shown). High mean *RF *distances for QILI (30.3%) and AvCon (8.1%), however, show that these methods fail to reconstruct reasonable trees from highly informative data sets with missing data. The mean RF distances for MinCut, SuperQP and SDM are between 1% and 2% and all remaining methods show an average *RF *distance of ≤1%.

### Bootstrapped phylogenetic trees

We extended the simulation with missing data (S, m, E, n) by bootstrapping the superalignment and the gene trees. In this case, reconstructed gene trees were the majority-rule consensus of trees reconstructed from bootstrapped alignments. Since branches with low support are discarded from each gene tree, the accuracy of supertree methods is expected to improve. Note that this bootstrap procedure does not a ect the medium-level methods. Here, we measured the accuracy of reconstruction for 200 of the alignments that were the basis of the simulations summarized in Figure 4b (S, m, E, n). The bootstrapped gene trees lead to an improvement of the accuracy of all supertree methods when compared to the results without bootstrapping (data not shown). The mean RF distance is now 5.6% for superalignment, between 9 and 10.3% for all MR methods, and between 12 and 22% for the cut methods.

### Gene-specific evolutionary rates (S, m, R*_α_*, n)

Now we introduce a more complicated setting where the evolutionary rates vary between genes. The true gene trees were generated from the species tree by stretching or shrinking all branch lengths with a Γ-distributed random factor drawn independently for each gene in each simulation. In two different settings, the shape parameter for the Γ-distribution was *α *= 3 and *α *= 1:67, respectively. As in the previous simulations, the substitution parameters for the sequence simulation were equal for each gene. The gene trees and the SA tree were also obtained by bootstrapping. For each choice of *α*, we computed 100 simulated alignments. For neither setting do the results differ substantially from the previous simulation with bootstrapping (data not shown).

### Gene-specific substitution parameters (S, m, P, n)

Here, as in the previous setting, the true gene trees differ from the species tree by their branch lengths. However, this time the branch lengths were fitted from the original data to obtain the true gene trees. For each alignment, the species tree was pruned to the respective taxon set. Afterwards, GTR parameters and branch lengths were fitted to the pruned tree using the original alignment. If a branch length got down to 10^-6^, the lower bound in IQPNNI, the respective branch length was set to 1/*l*, where *l *is the length of the corresponding alignment. The trees constructed this way were used as the true gene trees for the simulations. The sequence simulations used the estimated GTR parameters for each gene.

**Figure 6:**
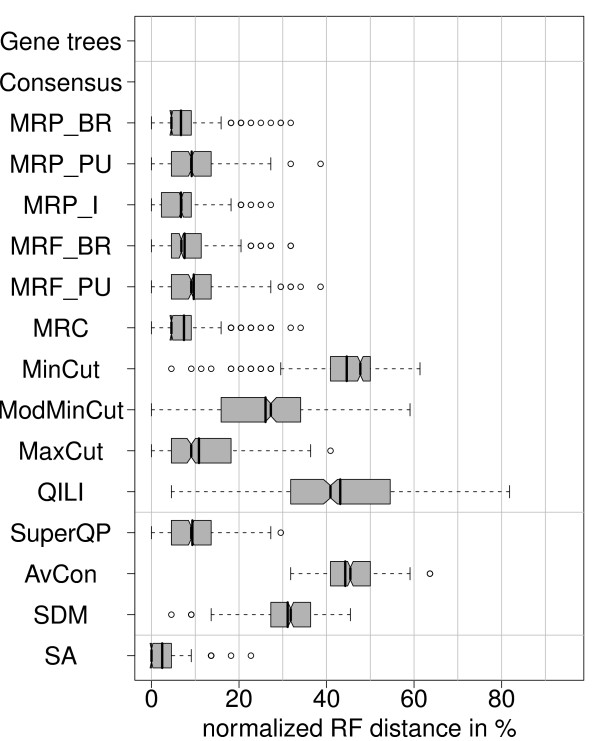
**Distribution of normalized RF distances (500 simulations) for the simulation setting S, m, P, n**. Simulation with gene-specific GTR parameters and missing data. The baseline distance is not applicable here (see text for details).

This simulation setting only allows for simulation of pruned data sets. Thus, the baseline distance is not applicable. The results cannot be compared directly to the previous simulations, since the average tree length is now larger, but the ranking of the methods can be compared. Figure 6 shows that the superalignment trees remain best (mean *RF *distance of 2.4%), even if simulation parameters differ between genes. SA, the MR methods, MaxCut and SuperQP are clearly better than the distance based methods, MinCut and ModMinCut.

### Gene specific topologies (S, m, G, n)

**Figure 7:**
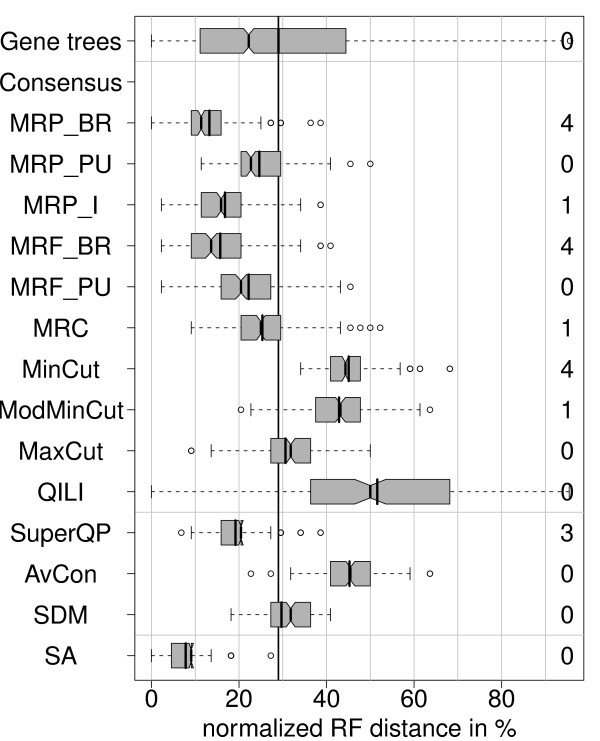
**Distribution of normalized RF distances (200 simulations) for the simulation setting S, m, G, n**. Simulation with gene-specific topologies and missing data. Note that the baseline distance is defined differently here: the gene tree distances are computed by comparing each reconstructed gene tree to the corresponding true gene tree and normalized with the appropriate number of taxa. The numbers on the right are the numbers of unresolved branches in the tree reconstructed from the original data with the corresponding method.

Here, the previous setting is extended as follows: Not only branch lengths and substitution parameters are gene-specific but also the topologies. Therefore, the gene trees reconstructed from the original data were used as true gene trees for this simulation. As before, only the setting with missing data can be studied, since the true gene trees already contain missing data. As we do not know the underlying species topology, a more complicated evaluation method is used: the inferred tree from each method is compared to the tree reconstructed from the true gene trees with the same method. e.g. an MRP_BR tree was reconstructed from the true gene trees and was used as a model tree when the distances to MRP_BR are evaluated in Figure 7. Also the early- and medium-level trees are reconstructed from the original sequence data and used for the distance computations. With this procedure, we estimate how consistently each method finds its own reconstructed species tree when sequence data are simulated along the gene trees. This is similar to a parametric bootstrap approach. Here, we face the problem that some trees reconstructed from the original data are not fully resolved. Also in these cases, we compute the Robinson-Foulds distances to these trees and normalize it with the same factor of 2(*n *- 3), where n is the number of taxa. Thus, the polytomies in these trees are treated as true and the distance increases if a tree reconstructed in the simulation is more resolved. To highlight this problem, we list the number of branches missing in the trees reconstructed from the original data on the right margin of Figure 7.

The resulting distances clearly show that SA is the most consistent method, since it has the smallest average distance to the SA tree from the original data (7.8%). It is followed by MRP_BR with a mean RF distance of 13.2%.

### Incomplete lineage sorting (S, c, T*_θ_*, e and S, m, T*_θ_*, e)

**Figure 8:**
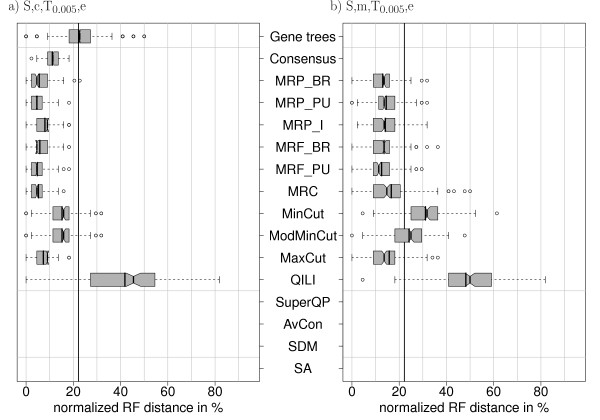
**Distribution of normalized RF distances (500 simulations) for the simulation settings S, c, T_0:005_, e and S, m, T_0:005_, e**. Simulation with gene-specific trees generated by a coalescent process (*θ *= 0:005) without reconstruction error. Early- and medium-level methods cannot be applied since no simulated sequences are available.

In this setting, the true gene trees were generated from the true model tree by a coalescent process (for details of the coalescent model used here, see Ewing et al. [67]). This can result in different branch lengths, but also different topologies. The species tree was rooted according to Figure 2b. From this rooted species tree, we simulated gene trees with different coalescent parameters. The coalescent parameter *θ *was used to generate incongruent gene trees with different amounts of incorrect branches. The larger *θ*, the more incongruence is caused by incomplete lineage sorting. e.g. *θ *= 0:005 results in a considerable incongruence among the gene trees: the mean normalized *RF *distance between the true species tree and the true gene trees is 22% (Figure 8a).

First, we investigate the performance of the supertree methods in the presence of incongruent gene trees without any reconstruction error. In Figure 8a, we see that the matrix representation methods can estimate the species tree quite accurately in the presence of complete data; MRP_PU and MRF_PU give the best results with a mean reconstruction error of 4.6% and 4.7%, respectively. The matrix representation methods, headed by MRF_PU (12.5%), are also the best methods when data are missing (Figure 8b).

### Incomplete lineage sorting and gene tree reconstruction (S, c, T*_θ _*, n and S, m, T*_θ _*, n)

**Figure 9:**
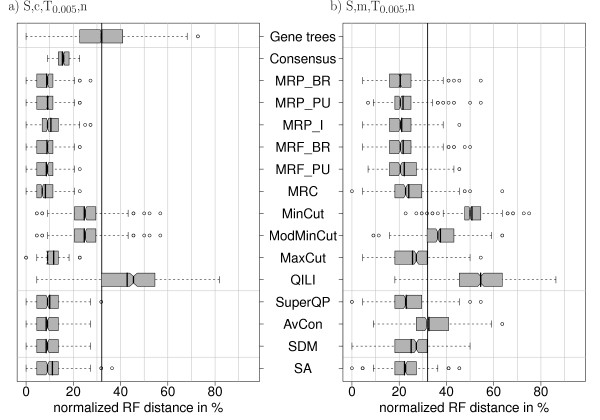
**Distribution of normalized RF distances (500 simulations) for the simulation settings S, c, T_0:005_, n and S, m, T_0:005_, n**. Simulation with gene-specific trees generated by a coalescent process (*θ *= 0:005). The true gene trees are equal to the trees used in Figure 8. Now, alignments are simulated along these trees and ML trees are reconstructed.

The gene trees from the previous section are taken as true gene trees. Along these, sequences are simulated and phylogenies are inferred as before. Thus, reconstruction error is added to the error present due to incomplete lineage sorting. The mean distance of the inferred gene trees to the species tree is 32% (Figure 9a). In the case of complete data, this distance is decreased by all methods except QILI. The distributions and mean distances of MRP_BR (8.7%), MRP_PU (9.1%), MRP I (10.5%), MRF_BR (8.9%), MRF_PU (8.6%), MRC (8.2%), MaxCut (11.7%), SuperQP (10%), AvCon (8.5%), SDM (8.5%) and SA (11.1%) are very similar. Thus the differences between the methods are less distinct. However, the mean superalignment distance is now larger than the average distances of most methods.

This might be due to the small number of genes (10) and the different sequence lengths (Figure 2a). More than 50% of all positions in the superalignment stem from only three genes. The corresponding three inferred gene tree topologies also show the smallest average *RF*-distances to the superalignment tree (numbers not shown). Thus these three genes mainly drive the superalignment reconstruction. If their gene trees are distant from the true species tree, the superalignment result will also deviate.

We also tested the methods on incongruent gene trees together with missing data. That is, the same alignments were used but the information was pruned according to Figure 2a. Several methods show a lower mean accuracy than the phylogeny of a full gene, namely MinCut, ModMinCut, QILI and AvCon (Figure 9b). MRP_BR (20.4%), MRP_PU (21.6%), MRP_I (21.1%), MRF_BR (21.7%) and MRF_PU (22.2%) still outperform superalignment (22.3%) on average, but the difference is marginal.

**Figure 10:**
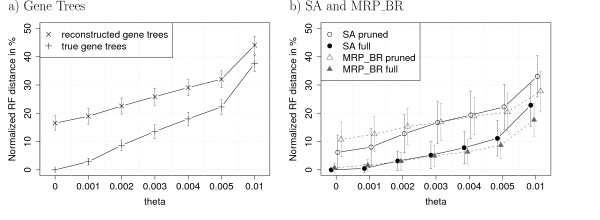
**Mean and standard deviation of the RF distances with different levels of incongruence**. The results of the true and reconstructed gene trees are computed from the distribution of mean distances of all simulations. Note that *θ *does not increase linearly but the last step is a doubling. The detailed results for *θ *= 0 and *θ *= 0:005 are shown in Figure 4 and Figure 9, respectively. Different numbers of simulation replicates were used: 500 for *θ *= 0 and *θ *= 0:005 and 200 for the remaining settings.

However, the above behavior is not representative for all degrees of incomplete lineage sorting. In Figure 10a, we see how the mean normalized RF distance of the true gene trees to the true species tree increases with *θ*. As a consequence, the distances of the reconstructed gene trees increase, too. At low *θ *(0.001-0.002), the reconstruction error exceeds the error introduced by incomplete lineage sorting. In this parameter area we observe figures similar to Figure 4 with SA performing better on average (figures not shown). With very high *θ*, however, the error introduced by incomplete lineage sorting is larger than the reconstruction error added to the true gene trees. In this parameter area, we observe that MRP_BR slightly outperforms SA (Figure 10b). MRP_BR is used here as a representative supertree method, which usually performs well compared to other methods.

Note that in each case, the standard deviations are overlapping with the mean of the competing method (Figure 10b). However, we must keep in mind that the data are paired, i.e., for each of the 500 simulations with *θ *= 0, we get one distance value for SA and one for MRP_BR. Thus, we tested the null hypothesis that the median difference in these paired distances is 0 using the Wilcoxon signed-rank test (Table 4). The results shown in Table 4 support the conclusion that SA is significantly better in regions where the error introduced by phylogenetic reconstruction is prevalent, whereas MRP BR is significantly better in regions where true gene trees differ a lot. Thus, if the reconstruction error dominates the error caused by incomplete lineage sorting, SA is the most accurate method by minimizing stochastic error. On the other hand, if incomplete lineage sorting is the prevalent source of gene tree incongruency, reconstructing the trees first and then applying a supertree method is favorable. However, in the case of high incomplete lineage sorting effects, the accuracies of all reconstruction methods are quite low. Figure 9 shows that about 8% of the branches are reconstructed incorrectly with complete data and about 20% with missing data for the best reconstruction methods.

**Table 4:** Paired Wilcoxon signed-rank test

		Complete data			Missing data		
			
*θ*	Sample size	*p*-value	Median difference	Confidence Interval	*p*-value	Median difference	Confidence interval
0	500	<**2.2 **× **10^-16^**	**1.5**	[1.5, 1.5]	<**2.2 **× **10^-16^**	**2.5**	[2, 2.5]
0.001	200	**2.2 **× **10^-9^**	**1.5**	[1, 1.5]	**2.2 **× **10^-16^**	**2.5**	[2, 2.5]
0.002	200	0.69	1.8 × 10^-5^	[-0.5, 1:9 × 10^-5^]	**2.2 **× **10^-6^**	**1**	[0.5, 0.5]
0.003	200	0.6	2.478 × 10^-5^	[-0.5, 0.5]	0.69	3.2 × 10^-5^	[-0.5, 0.5]
0.004	200	**1.8 **× **10^-3^**	**-1**	[-1.5, 3:2 × 10^-5^]	0.47	-6 × 10^-5^	[-0.5, 0.5]
0.005	500	**1**:**6 **× **10^-14^**	**-1.5**	[-1.5,1]	**1.1 **× **10^-8^**	**-1**	[-1.5, -0.5]
0.01	200	**6**:**1 **× **10^-16^**	**-2.5**	[-3,-2]	**7.4 **× **10^-15^**	**-2.5**	[-3, -2]

The distances of MRP)_BR are compared with SA, thus positive median differences stand for higher distances in MRP_BR and negative differences stand for higher distances in SA. *p*-values < 0:05 find median differences whose 95%-confidence intervall does not include 0 are marked in bold.

## Conclusions

We presented a detailed simulation study to assess the accuracy of superalignment, supertree and medium-level methods for reconstructing phylogenetic trees from multiple data sets. Although supertrees are often used to combine data of different kinds, our simulations only refer to sequence-based approaches. Morphological characters are not included due to the lack of reasonable probabilistic models to simulate their evolution. This study is first in comparing a broad range of methods for combining incomplete data sets. Furthermore, the true gene trees were generated from the true species tree in different ways (see also Table 3): (a) all gene trees were identical to the species tree, (b) the branch lengths but not the topology were gene-specific, (c) the gene trees from the original data were used as true gene trees and (d) the gene trees showed different topologies modeling incomplete lineage sorting. All conclusions are based on the specific implementations used for these methods as described in the methods section.

Gene features like sequence lengths and taxon overlap influence the accuracy of the methods presented. Instead of covering many different parameter combinations, we used the parameters of two very different natural data sets for the simulations. They cover 10 genes of 25 taxa and and 254 genes of 69 taxa, respectively. Note that supertree methods can be applied to substantially larger data sets (e.g. [20]). We expect that the accuracy of the methods will be influenced by the amount and distribution of missing data as well as the taxon overlap between alignments. Additionally, the incongruency among the true gene trees and alignment lengths influence the relative performance of the methods. Adding more genes may increase the number of incongruent trees [68], while adding more taxa typically increases the amount of missing data. Thus, accuracy will generally decrease.

The first main result is that one of the matrix representation methods, which are the most abundant supertree reconstruction methods used in the literature, usually shows the second-best result after superalignment. Especially the MRP and MRF methods with Baum/Ragan-coding result in very accurate trees. Since these methods are based on splits, bootstrap-based weighting can be easily incorporated, which is expected to further increase the accuracy of the reconstructed trees [28,32]. Among the medium-level methods, SuperQP yields better results than the distance-based approaches, especially when data are missing. The accuracy of SuperQP is often consecutive to or among the accuracies of the MR-based supertree methods.

Second, in the case of complete gene trees, the majority-rule consensus method is also applicable. In all simulation settings with complete gene trees, some supertree methods perform better on average than the consensus method. In these cases, supertree branches that are supported by less than half of the gene trees are correctly resolved, while remaining unresolved in the consensus tree. This shows that, although supertree methods have been criticized for not being majority-rule methods [69], the resolution of additional branches can be favorable. However, as for majority-rule consensus trees, it is desirable to also label the supertree branches with the support in the gene trees.

Third, we introduced the baseline distance as a measure to judge the benefit of the combination methods. The baseline distance for one setting is defined as the mean *RF *distance between the true species tree and the reconstructed gene trees using complete alignments. We observe that, for most of the simulation parameters studied here, QILI, average consensus, MinCut and modified MinCut show larger mean *RF *distances than the single gene trees. QILI has already been observed to be slightly worse than MRP [58]. Average consensus is clearly outperformed by SDM when data are missing. We applied both methods as medium-level methods by taking pairwise distances directly from the alignment distances, not from the reconstructed gene trees. While average consensus was suggested as a late-level method [14], SDM has been explicitly designed as a medium level method [15]. Thus, average consensus may not be able to resolve the conflicts in the non-tree-like distances. The behavior of MinCut can be partly explained by the fact that it resembles Adams consensus [54]. This means that uncertain taxa will be placed at the root of subtrees, which can disturb quite a few splits, leading to high *RF *distances. The cut methods presented here implement a heuristic based on the rooted triplets in the gene trees. Recently, Lin et al. [70] suggested another approach which maximizes the common rooted triplets in the supertree and the gene trees. They show that their method outperforms modified MinCut and MaxCut on example data sets.

Finally, we observe that superalignment methods usually show the highest accuracy on average. This applies to incomplete data as well as gene-specific substitution parameters. Superalignment also results in the most consistent phylogenetic estimation when each method is not compared to a model tree but to the original result obtained with that very method (Figure 7). However, in the presence of high incongruency among true gene trees, that means, if reconstruction error is not the main cause that gene trees differ from the species tree, the implicit weighting by sequence length can have a negative effect on the performance of superalignment leading to outperformance by the supertree method MRP BR. This bias might be avoided by introducing a normalization, but then, the opposite and still unwanted bias could emerge. Furthermore, it has been discussed (e.g. [71]) that SA should be preferred over supertree methods since the former does not imply character weighting. Furthermore, Edwards argued recently [72] that in the presence of gene tree conflict caused by coalescence effects as many genes as possible should be used and they should be weighted equally. This is consistent with our observation, that supertree methods outperform superalignment in the presence of strong coalescence effects. There are also species tree reconstruction methods that use a coalescent model to account for the differences between true gene trees (e.g. [73]). Kubatko et al. [74] have shown that concatenation of gene alignments may be inappropriate when the gene tree histories differ considerably. The coalescent model can be applied for closely related species (e.g. grasshoppers [75]), but severe problems caused by incomplete lineage sorting seem not to play a role among taxa of deep phylogenetic trees (e.g. for Metazoa [67]). Since these methods typically require complete data, we did not include them in our comparison. We rather concentrated on methods which were explicitly designed for missing data and that resolve conflict of unknown source.

Our results are in general concordance with previously published comparisons. Dutilh et al. [7] used real data sets and also found superalignment to perform best. Eulenstein et al. [34] used simulated data and find MRP and MRF to perform similar and better than MinCut and ModMinCut. Swenson et al. [76] compare superalignment and weighted and unweighted MRP using biologically motivated simulations and also find the highest accuracy for superalignment. We apply, however, a broader range of methods than previous studies.

All conclusions presented here are based on the accuracy measured by the mean *RF *distance. This does not imply that the methods presented as better on average always show superior results and could, thus, be used as a gold standard. Rather, we highly recommend to use several of the superior methods (considering also various levels of data combination) and to compare their results. By comparing the accuracies of the reconstructed supertrees with the accuracies of the ML gene trees, we showed the baseline distance to be a reasonable criterion for excluding unsuitable methods. If the baseline distance cannot be improved by a data combination method, it is preferable to use only genes for ML reconstruction that are present in all taxa and to possibly sequence the missing genes in some taxa. For a real data supertree analysis, not the baseline distance but only the distances between the reconstructed gene trees are available to assess which method may be appropriate. Thus, the homogeneity of the gene trees can be an indicator whether variation is present in the gene trees. Assessing the homogeneity of overlapping gene trees is a complex task of itself [23,77-79] and is not covered in our study.

The source of variation, i.e., why the reconstructed gene trees differ from the species tree, should also be taken into account, since it has an influence on the relative performance of the methods. If a tree-like evolutionary history is assumed and true gene tree incongruence is unlikely or rare, superalignment results in the most accurate trees. This also holds in the presence of gene-specific substitution parameters and branch lengths, as has been observed before [27]. But if the difference of the true gene trees to the species trees is the main source of variation, supertree methods are favorable. Applying a superalignment method to data with different underlying topologies or highly varying parameters has also been shown to be problematic (e.g. [80,81]).

In the case of known gene tree variation, methods that model the assumed causes can also be applied (e.g. [67,82] for incomplete lineage sorting). When exploring gene tree effects, like horizontal gene transfer or incomplete lineage sorting, gene trees have to be reconstructed and compared to a species tree. If the intention of an analysis is species tree reconstruction, however, external information may be considered: External information, like the rates of horizontal gene transfer, gene duplication or incomplete lineage sorting helps to judge whether complex evolutionary models are necessary to reconstruct the species tree. If these complex scenarios are not assumed to play a major role, application of superalignment minimizes the stochastic error. On the other hand, if gene-tree conflict is present but the underlying biological model is unknown, supertree or medium-level methods may be more reasonable. They account for gene tree variation but make no assumptions on the underlying evolutionary model causing the variation.

Our study provides comparative data for methods combining the data at different levels. This broad collection of methods hence provides valuable help to choose a promising set of approaches to reconstruct species trees from sets of orthologous genes.

## Availability and requirements

A Software to simulate data as in this study is available from our webpage http://www.cibiv.at/software/supi. The software published under GPL is written in Python and Java and is platform independent.

## Competing interests

The authors declare that they have no competing interests.

## Authors' contributions

AK carried out the simulations and prepared the manuscript. AvH and HAS designed the study, discussed the results and contributed to the manuscript. All authors read and approved the final manuscript.
